# Microbiota Associated with Infections of the Jaws

**DOI:** 10.1155/2012/369751

**Published:** 2012-07-08

**Authors:** Elerson Gaetti-Jardim, Luis Fernando Landucci, Kathlenn Liezbeth de Oliveira, Iracy Costa, Robson Varlei Ranieri, Ana Cláudia Okamoto, Christiane Marie Schweitzer

**Affiliations:** Laboratório de Microbiologia e Imunologia, Departamento de Patologia e Propedêutica Clinica, Faculdade de Odontologia, UNIV-Universidade Estadual Paulista, Araçatuba, Brazil

## Abstract

The microbial infections involving the craniofacial skeleton, particularly maxilla and mandible, have direct relationship with the dental biofilm, with predominance of obligate anaerobes. In some patients, these infections may spread to bone marrow or facial soft tissues, producing severe and life-threatening septic conditions. In such cases, local treatment associated with systemic antimicrobials should be used in order to eradicate the sources of contamination. This paper discuss the possibility of spread of these infections and their clinical implications for dentistry, as well as their etiology and aspects related to microbial virulence and pathogenesis.

## 1. Introduction

Infections of the of the craniofacial skeleton, especially maxilla and mandible, are common and their etiology is associated to oral microbial biofilm and other oral microbial communities [1, 2]. Their prevalence is significantly higher in developing countries, with poor socioeconomic conditions, and where standards of oral hygiene and health are unsatisfactory [2, 3]. Although most orofacial infections are sensitive to a clinical and antimicrobial treatments, in some patients, these infections may be refractory, and these cases demand a multidisciplinary approach [2, 4, 5].

The most relevant infections of the jaws are represented by lateral and apical periodontitis, osteomyelitis, peri-implantitis, and their complications, such as facial cellulitis and other infections involving deep spaces of face and neck. The treatment of these conditions involves local procedures and, sometimes, specific antimicrobial drugs [4] and microbial resistance to most common drugs is widely disseminated, the microbial diagnosis of such infections is relevant to the clinician [4, 6].

Thus, the objective of this paper is to present some data about microbial etiology of the most common infections of the maxilla and mandible and their significance in dentistry.

## 2. Chronic Periodontitis

Lateral periodontitis is a biofilm-associated inflammatory disease of the periodontium and constitutes the most frequent cause of early tooth loss in adults [7]. It is clear that initiation of lateral periodontitis and its progression are multi-factorial, involving microbial aggression, host mechanisms, environmental conditions, and complex interactions among all these elements [8]. Recently, chronic lateral periodontitis has been understood as a consequence of a microbial shift in the composition of dental biofilm, with reduction of beneficial symbionts and an increase in the number of pathogens [7].

The microbiota associated with lateral periodontitis has been intensely studied in the last decades and our knowledge about etiological factors involved in the establishment and progression of this condition is very limited, which is aggravated by the fact that a significant portion of the periodontal microbiota remains uncultivable [9]. In addition, most of periodontal microbiota is present in dynamic and complex biofilms, which are influenced by several local and systemic factors, such as host-mediated selective pressure, age, tobacco and drug consumption, antimicrobials, availability of iron, and presence of orthodontic appliances, prosthesis, and restorations, affecting biofilm retention [8]. Ethnic and geographic particularities seem to interfere with the composition of microbial biofilm associated with lateral periodontitis [10–12].

Lateral periodontitis is primarily caused by complex consortia of microorganisms [7, 13, 14]. However, the presence of such microorganisms does not imply the development or progression of this infectious process since significant part of human population harbor them without any clinical or radiographic evidence of periodontal bone or attachment loss [3, 13, 15–17]. In periodontally healthy patients, the putative periodontopathogens are present in low numbers as members of resident microbiota and they contribute to the maturation of human adaptive immune system and comprise part of innate defense mechanisms of oral cavity [18, 19].

Only a few microbial species have been strongly implicated as etiological agents for chronic and aggressive forms of periodontitis and each microbial species presents its virulence within a consortium, not acting independently of their neighbors, in a very complex microbial community in the oral biofilm. The group of most relevant and studied cultivable species associated with periodontitis is composed of *Porphyromonas gingivalis*, *Aggregatibacter actinomycetemcomitans*, *Prevotella intermedia*, *Tannerella forsythia*, *Campylobacter rectus,* and fusobacteria [3, 14, 15, 20], but the use of culture-independent methods to detect disease-associated subgingival microorganisms has completely changed this view [21].

The pathogenicity of different strains of selected subgingival microorganisms seems to vary widely. For example, the JP2 clone of *A. actinomycetemcomitans* has a 530 base pair deletion in its leukotoxin reporter gene operon that exacerbates production of leukotoxin by 10–20-fold compared to minimally leukotoxic strains [11, 22], and this clone is common in aggressive cases of lateral periodontitis [11, 22–24]. The frequency of this genetic deletion in the JP2 clone is especially relevant, since subjects harboring highly leukotoxic *A. actinomycetemcomitans* are 22.5 times more likely to convert from healthy status to localized aggressive periodontitis than those colonized by strains harboring full-length leukotoxin promoter region [25]. Other studies have evidenced that clones of other periodontopathogens, such as *P. gingivalis*, also show extensive diversity in the virulence [26].

In aggressive periodontitis, besides highly leukotoxic *A. actinomycetemcomitans* and *P. gingivalis*, other microorganisms have been involved in periodontal destruction, such as *Parvimonas micra* and *Campylobacter rectus* [27], *Treponema lecithinolyticum* [28], and genus *Selenomonas* [21], but approximately 50% of the most prominent microbial taxa was not cultivable [21, 29]. The prevalence and populations of *P. endodontalis* and *Tannerella forsythia* are also significantly greater at periodontally diseased sites in aggressive or chronic forms of periodontitis [30].

The culture-independent studies have changed our comprehension about microbial ecology in periodontitis, and different bacteria appear to be associated with clinically similar periodontal status and the subgingival microbiota associated with disease establishment and progression may be person- and site-specific [1]. Therefore, the role of cultivable periodontopathogens must be reevaluated, since there is no single microorganism or microbial consortium that is always involved, even in aggressive periodontitis [31].

In the etiology of periodontitis, a possible role of microorganisms of the *Archaea* domain has been studied [32–35]. These microorganisms resemble bacteria, but they have different sequences in their DNA and are not in the *Bacteria* domain. Archaea domain has never been detected in the subgingival microbiota of periodontally healthy subjects [32, 34, 36] or at healthy sites in patients with periodontitis [32], and their populations in periodontally disease sites enhance as a function of disease severity [36]. However, the pathogenic potential of such microbes is under discussion [37]. Thus, the role of *Achaea* domain in periodontitis remains unclear, but it is possible that these microorganisms, particularly the methanogenic *Archaea*, contribute to aggression to periodontal tissues by means of interactions with other pathogens [38], affecting hydrogen and methane concentrations and the redox potential in gingival crevice or periodontal pockets.

## 3. Periapical and Endodontic Infections

Microorganisms reach the pulp through tooth fracture, dental caries, tubules of exposed dentine in the surface of root (result of fissures, radicular caries, and chronic periodontitis), anachoresis, and by dental procedures. The composition of primary endodontic infections reflects the oral microbiota and the method of dissemination. However, due to ecological interactions and environmental conditions, over time the oral anaerobes eventually become the predominant group in the endodontic and periapical infections [39–41], and the exposition of root canal to oral environment does not influence the predominance of such microorganisms in the apical portion of root canal system and periapical tissues [39]. 

The most prevalent microorganisms associated with primary endodontic infection are Gram-negative anaerobes, such as fusobacteria, genera *Porphyromonas*, *Selenomonas,* and *Prevotella*, followed by Gram-positive anaerobes of genera *Peptostreptococcus*, *Parvimonas,* and *Eubacterium* [40–43], generally associated with *Campylobacter* spp., *Eikenellacorrodens*, actinomycetes, and other microbial groups in complex biofilm structures [39, 42–44]. The vast majority of anaerobic species in these infections can also be recovered from the periodontal pockets [40, 43].

The presence of Gram-negative anaerobic bacilli and anaerobic Gram-positive cocci is associated with incidence of acute signs and symptoms, including pain, sensitivity on pressure, and swelling [45–47]. However, some studies did not find such correlations between the composition of microbiota and symptomatology [48].

Generally, presence of septic contents is limited to root canal system, but extraradicular presence of bacteria and yeasts has been evidenced, even after endodontic therapy [49, 50], particularly in cases refractory to treatment [51]. The presence of genera *Prevotella*, *Peptostreptococcus*, *Eubacterium*, *Propionibacterium* [45], and *Enterococcus* [52, 53] characterizes microbiota from secondary endodontic infections and refractory periapical lesions. Generally, microorganisms recovered from secondary endodontic and periapical infections are much more resistant to biomechanical procedures and antimicrobial agents used in the endodontic treatment and may be a source of opportunistic microorganisms [50].

## 4. Peri-Implantitis

Peri-implantitis is considered an inflammatory process affecting the tissues around dental implants, resulting in loss of peri-implantar bone [54].

Dental implants are currently a routine treatment. The implants are made of titanium or titanium alloy and may have its surface modified in roughness, as in chemically, for example, coated with hydroxyapatite [55]. In spite of the contamination of the surgical field where dental implants are placed, the clinical success rates are high, particularly when surgical and prosthetic procedures are correctly planned.

The goal of dental implants is the osseointegration of the titanium surface and vital bone, and this phenomenon, associated with the union of titanium oxide with gingival tissues, diminishes the invasion of oral microorganisms, preventing the occurrence of peri-implantitis [55], although this phenomenon is not sufficient to ensure long term implant survival and cases of peri-implantitis [56].

However, no single microorganism has been closely associated with peri-implantitis, which have characteristics of typical mixed infections [56, 57]. The occurrence of bone loss around dental implant varies from 5% to 56% of dental implants [58, 59], involving 12%–43% of implant sites [59].

It has been demonstrated that there is a significant change from a predominately Gram-positive facultative anaerobic microbiota towards a biofilm with a greater proportion of Gram-negative, strict anaerobes at peri-implantar sites with inflammation [60, 61]. The composition of microbial biofilm associated with peri-implantitis and failing dental implants is similar to subgingival microbiota involved with lateral periodontitis; with a large predominance of strict anaerobes, particularly genera *Porphyromonas*, *Prevotella*, *Tannerella*, *Parvimonas*, and *Fusobacterium*, associated with facultative bacteria [62, 63]. 

These microorganisms are capable to invade peri-implant tissues and induce inflammation, which may result in eventual implant failure. In addition, microorganisms not usually associated with lateral periodontitis or odontogenic abscesses such as staphylococci, enteric Gram-negative bacteria, enterococci, and yeasts are commonly detected in peri-implant infections [56, 62, 64]. The isolation of staphylococci from peri-implant infection is significant as both coagulase-positive and coagulase-negative staphylococci are frequently responsible for infections associated with metallic biomaterials and in-dwelling medical infections in general [65], and this Gram-positive cocci are able to adhere and colonize titanium surfaces, what can be relevant in subsequent infections around dental implants and in the etiology of peri-implantitis [66, 67].

In some patients who present severe bacterial growth on implant surfaces pseudomonas and members of family *Enterobacteriaceae* are detected by culture and culture independent methodologies [56], and these pathogens are frequently resistant to antimicrobials and involved in the majority of nosocomial infections. However, the role of *Enterobacteriaceae* in chronic periodontitis remains unclear, and they are thought to indicate superinfection or opportunistic infection in immunosuppressed or irradiated patients [68].

In spite of role of major periodontopathogens in the etiology of lateral periodontitis, some species seem to not actively participate in peri-implantitis, such as *A. actinomycetemcomitans* [56, 60, 63]. In general, most oral anaerobes have the potential to be in increased numbers in peri-implantitis, but the name of the most prominent species varies from patient to patient, as previously discussed for lateral periodontitis. 

## 5. Chronic Osteomyelitis

Osteomyelitis of the maxillofacial skeleton, especially involving maxilla and mandible, may be acute or chronic in nature, but almost all cases are chronic and associated with dissemination of odontogenic infections [2]. The occurrence of such infection is not common in developed countries. However, its occurrence is significant in communities with poor socioeconomic conditions and populations with poor standards of oral hygiene. These infections can be limited to a single anatomic site or spread to other areas of the bone marrow, bone tissues, or even adjacent soft tissues, particularly in individuals with immunosuppression or metabolic impairment, such as noncontrolled diabetics, hospitalized and symptomatic HIV+ patients.

Although osteomyelitis may be either of hematogenous origin or adjacent to infectious processes [69, 70], in the jaws, most cases are associated with endodontic infections [69], peri-implantitis, lateral periodontitis, and gingivitis [71].

The microbiological aspects of osteomyelitis of the jaws are unclear [69]. However, the composition of the microbiota associated to the chronic osteomyelitis of the jaws is influenced by the origin of the infectious process [72]. In hematogenous origin, it is notorious the relevance of oxygen tolerant microorganisms, such as enteric rods and staphylococci, meanwhile in the chronic osteomyelitis associated with previous odontogenic infections, the etiology of the osteomyelitis will depend on the microbiota of the previous infectious process and where it took place, generally producing mixed infections with predominance of oral anaerobes, particularly genera *Fusobacterium*, *Porphyromonas*, *Prevotella*, *Parvimonas*, and *Eikenella* frequently associated with actinomycetes and staphylococci [2]. In these cases of chronic osteomyelitis of the maxilla and mandible, the major source of the infecting microorganisms is odontogenic, producing an average of 2.4 to 3.9 strict anaerobic species and 0.4 to 1.5 aerobic or facultative anaerobic species [73].

Studies have evidenced that misuse of antimicrobial drugs and history of trauma may facilitate the implantation of oral facultative anaerobes and enteric rods and cocci, especially *Enterococcus faecalis* in chronic osteomyelitis of the jaws, even in cases associated with chronic periodontitis or periapical periodontitis [72, 74].

The predominance of oral anaerobic species in mixed infections in bone marrow suggests that the ecological associations are relevant in the development of osteomyelitis of the jaws. Since most oral microorganisms are able to co-aggregate in biofilms, treatment of the chronic infections involving bone marrow of the jaws frequently involves removal of bone sequestrations, curettage, debridement, and systemic antimicrobials for weeks [75].

The role of anaerobes in the progression of chronic osteomyelitis has suggested the use hyperbaric oxygen as an adjunct in treatment of such bone infections, in association with combined antibiotic and surgical approach. This approach is particularly efficient in refractory cases, particularly in immunosuppressed and diabetic patients [76]. In addition, the stimulation of angiogenesis around the healthy tissues produced by action of the hyperbaric oxygen may reduce or prevent tissue invasion [2].

## 6. Facial Cellulitis of Dental Origin

This condition is defined as subcutaneous acute infection around the mandibular and maxillary bones [77], and due to this relationship with skull bones, it was included in this paper.

Facial cellulitis of dental origin affects patients of all ages, but they are commonest in younger patients [77] and disseminates from the primary dental infections through the subperiosteum area, and from this area it breaks the bone structures of the jaws, invading soft tissues of the face. The data about etiology of such infections are scarce, but oral strict anaerobes seem to be predominant [77]. This infection is polymicrobial in 75% of the cases, producing from two to six bacterial species per clinical specimen [77–79], and the most prominent microbial association is between Gram-positive aerobic-facultative aerobic cocci and Gram-negative anaerobic bacilli [79].

The literature has evidenced that black-pigmented *Prevotella*, *Porphyromonas* spp., *Bacteroides* spp., and *Fusobacterium* spp. are usually present [77, 78, 80], followed by other microorganisms, such as *Eubacterium* spp., *Veillonella* spp., oral streptococci, and staphylococci [77].

The role of such facultative anaerobes and aerobes in these infections is not clear, but it is possible that they may create favorable conditions for strict anaerobes due to oxygen consumption and their invasive potency on local tissue, reducing the redox potential of colonized tissue. The same mechanism may be involved in the ecological succession in endodontic-periapical infections and other oral infectious diseases.

## 7. Lemierre's Syndrome

This infection is a rare but severe life-threatening complication of oral infections, resulting in lateral pharyngeal space infection [81]. It is characterized as thrombosis and suppurative thrombophlebitis of the internal jugular vein with dissemination of septic emboli to the lungs and other sites [81, 82]. Before the antimicrobial era, death was the common result. The microbiota of such complication remains uncharacterized, but fusobacteria, in particular *F. necrophorum*, *F. nucleatum*, *F. gonidia forum,* and *F. varium*, are commonly detected [81]. Genera *Prevotella*, *Bacteroides*., and *Peptostreptococcus* are also involved in the pathogenesis. The occurrence of methicillin resistant *S. aureus* in Lemierre's syndrome has been described [83, 84], but the casuistic is not big enough to determine the role played by these cocci in the etiology of the infection, but illustrates a significant increase in the incidence of these Gram-positive microorganisms in head and neck infections [85].

## 8. Deep Head and Neck Infections

The most frequent and serious complication of dental infections involve dissemination of bacteria to deep neck structures and submandibular and sublingual spaces, which are enclosed by the deep cervical fascia. The anatomical sites among these fasciae represent areas of loose connective tissue filling and the spaces of the neck communicate with one another forming channels by which infections may spread over large areas [86].

Deep space head and neck infections are among the most frequent clinical conditions within the maxillofacial area and mostly require an accurate surgical and antimicrobial treatment [87]. Maxilla and mandible are the most common origins of these serious and life-threatening infections in Dentistry [4, 86, 87]. Generally these conditions are associated with microbial dissemination of oral bacteria from chronic periodontitis, periapical infections, or the overgrowth of microorganisms of the dental biofilm [86–88]. The classical deep neck infection is the Ludwig's angina, involving a bilateral infection of both the submandibular and sublingual spaces.

A serious concern about these infections is the diagnosis, which may be delayed due to previous use of antimicrobials, because the drugs may mask local signs, such as swelling. Other clinical signs and symptoms include fever, pain, lymphadenopathy, odynophagia, dysphagia, and trismus [88, 89].

These infections are mixed aerobic-anaerobic infections [89]. Oral anaerobes, particularly genera *Porphyromonas*, *Fusobacterium*, *Prevotella*, *Peptostreptococcus*, and facultative oral streptococci, frequently associated with enteric microorganisms, are the commonest pathogens that cause deep neck infections [4, 5, 86, 90, 91]. More than two-thirds of deep neck infections contain *β*-lactamase-producing organisms, which may affect the efficacy of antimicrobial agents [86]. In cases of fistulization of neck infection, coagulase-negative staphylococci are the most frequently involved [86] and some strains are methicillin-resistant [5, 85]. In some cases, unsuspected microorganisms may be present, such as pseudomonas and members of the *Burkholderia cepacia* group [89].

Natural evolution of such conditions, without treatment, may lead septicemia or, empyema, airway collapse, or descending mediastinitis [4, 89, 90].

## 9. Conclusions

This overview of infections in the jaw illustrates the great variety of microorganisms involved, and most of such diseases are polymicrobial in nature. Usually, these microbes originated from the dental biofilm and spread towards periodontal and periapical tissues, reaching the spaces of the face and occasionally evolving to systemic sepsis. In these severe cases, with involvement of bone marrow or life-threatening infections, such as facial cellulitis or Ludwig's angina, antimicrobial agents should be associated with local treatment, which generally aims eradicate the source of invading microorganisms.
